# Real-time estimation of respiratory rate from a photoplethysmogram using an adaptive lattice notch filter

**DOI:** 10.1186/1475-925X-13-170

**Published:** 2014-12-17

**Authors:** Chanki Park, Boreom Lee

**Affiliations:** School of Mechatronics, Gwangju Institute of Science and Technology (GIST), Gwangju, South Korea; Department of Medical System Engineering (DMSE), Gwangju Institute of Science and Technology (GIST), Gwangju, South Korea

## Abstract

**Background:**

Many researchers have attempted to acquire respiratory rate (RR) information from a photoplethysmogram (PPG) because respiration affects the waveform of the PPG. However, most of these methods were difficult to operate in real-time because of their complexity or computational requirements. From these needs, we attempted to develop a method to estimate RR from a PPG with a light computational burden.

**Methods:**

To obtain RR information, we adopt a sequential filtering structure and frequency estimation technique, which extracts a dominant frequency from a given signal. In particular, we used an adaptive lattice notch filter (ALNF) to estimate RR from a PPG along with an additional heart rate that is utilized as an adaptation parameter of our method. Furthermore, we designed a sequential infinite impulse response (IIR) notch filtering system (i.e., harmonic IIR notch filter) to eliminate the cardiac component and its harmonics from the PPG. We compared the proposed method with Burg’s AR modeling method, which is widely used to estimate RR from a PPG, using open-source data and measured data.

**Results:**

By using a statistical test, it was determined that our adaptive lattice-type respiratory rate estimator (ALRE) was significantly more accurate than Burg’s AR model method (p <0.0001). Furthermore, the ALRE’s tracking performance was better than that of Burg’s method, and the variances of its estimates were smaller than those of Burg’s method.

**Conclusions:**

In short, our method showed a better performance than Burg’s AR modeling method for real-time applications.

## Background

The photoplethysmogram (PPG) is one of the bio-signals that can be acquired using a pulse oximetry sensor placed on a finger or ear lobe to measure O_2_ saturation. In addition, it can measure other physiological information such as pulse rate (or heart rate) and respiratory rate (RR) by one wearable sensor unlike electrocardiography [1]. Therefore, mobile healthcare system often utilizes a PPG sensor to acquire several kinds of health information including RR simultaneously in a simple module [2]. The pulse oximetry sensor is composed of an infrared (or red) transmitter and receiver, and these two devices are mounted on both sides of the target subject (finger or ear lobe). This sensor measures a transmitted light intensity from the transmitter to the receiver, and its measured value indicates an absorbance of the light in tissue and blood. In particular, the absorption of infrared light (absorbance) is proportional to material characteristics such as molar absorptivity, molar concentration, and path length. This principle is called the Beer–Lambert law [3].

**Figure 1:**
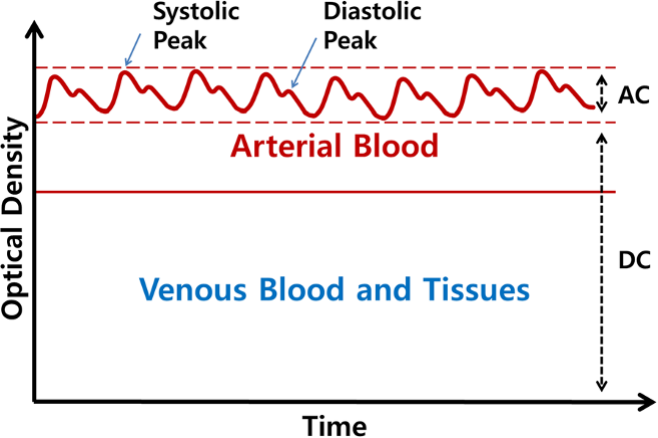
**Composition of PPG.** PPG wave is generated by fluctuating volume of arterial blood.

The modulation of PPG induced by breathing has not been fully understood. Nonetheless, the fluctuation of blood volume in the peripheral vascular bed caused by respiration is already well known [4] and was modelled by three modulation types [5]. As a result, the acquisition of a respiratory component from PPG is possible because PPG reflects the blood volume changes. Usually, a PPG consists of AC and DC components. The AC signal represents the absorbance of pulsed arterial blood, and the DC signal indicates the absorbance of non-pulsed blood and tissues (Figure 1). In this study, we used the AC signal component of a PPG to estimate the respiratory rate.

Vital signs, which consist of heart rate (HR), respiratory rate, blood pressure, and body temperature, have long been used as basic information in healthcare systems [6]. For example, pulmonary or cardiovascular diseases can be detected by measuring RR [7]. In our study, we focused on the RR information that is merged in a PPG signal, and estimated the HR for use in the RR estimation. HR is easily obtained not only by counting the number of zero crossings or peaks of the PPG [8], but also by analyzing the frequency of the PPG signal’s cardiac component, which is sufficiently large to estimate HR. However, it is difficult to estimate RR from a PPG because the respiratory component of the PPG is not clearly observed in the signal.

Because of the physiological response of the cardiopulmonary system, respiration induces three modulations in a PPG: amplitude, baseline, and pulse width modulations [5]. From the presence of the respiratory response in a PPG, many researchers have been motivated to develop or utilize methods for RR estimation from a PPG, such as digital filters, the autoregressive (AR) model, variable frequency complex demodulation, and particle filters [2, 8–14]. Nakajima et al. used digital filters to estimate HR and RR from a PPG, but this method required specific ranges of HR and RR. For example, RR should be less than 0.6 Hz [8]. Fleming and Tarassenko suggested a method to estimate RR from a PPG using the autoregressive model, and its estimate was considerably accurate. Because autoregressive modeling requires batch processing, they used a moving-window method for the real-time process [10]. The computational efficiency of the AR model method was considerably reasonable, but for real-time applications, it had an inefficient structure caused by the overlapped moving window method. Recently, AR modeling for HR and RR estimation was proposed for video-based vital sign monitoring [11]. Chon et al. suggested a high resolution time frequency analysis for RR estimation from a PPG [12, 13]; the method was called the VFCDM. This method showed outstanding accuracy, but its computational burden was not sufficiently light to construct a real-time monitoring system [5, 14].

**Figure 2:**
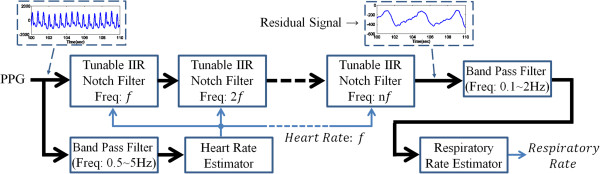
**Overall system.** The ALRE is constructed by two frequency estimators and harmonic IIR notch filter.

The need to design a comfortable, portable, and fast-processing system has grown stronger as ubiquitous healthcare industry has grown [15]. Although many researchers have achieved a technical progress to monitor the cardiopulmonary system [1, 16, 17], mobility and fast processing remain as challenges [15]. Because of these challenges, we set two conditions for designing a RR estimator from a PPG as follows: (1) light computation, and (2) on-line processing. In order to satisfy these conditions, we propose an adaptive filter structure that combines a sequential infinite impulse response (IIR) notch filter to remove the harmonic components of the heart rhythm, and two on-line frequency estimators (see Figure 2). Especially, we adopt an adaptive lattice notch filter (ALNF) for the frequency estimator [18, 19].

In the next section, we explain the relationship between the AR modeling method and our approach, the structure of the proposed algorithm, and the ALNF, which serves as a frequency estimator in our method. Then, we compare the proposed system with Burg’s AR method using experimental results. Finally, we discuss the results and future works.

## Methods

A PPG can be modulated by the respiratory activity in three manner: baseline trend, amplitude, and pulse width modulations [2, 5]. To design an estimation system that uses simple processes, we assumed that the modulations in a PPG caused by respiration can be simplified by only a baseline modulation without amplitude and pulse width modulations, which is suitable in most real-life situations. From this assumption, we can model a PPG by adding cardiac and respiratory components as follows:
1

*v* (*n*): white Gaussian noise

*w*_o_: *heart rate*, *w*_1_: *respiratory rate*

where the first term represents the cardiac component and the second term does the respiratory component.

In this study, we suggest an adaptive algorithm to estimate respiratory rates from a PPG in real-time. This algorithm is constructed by simple methods that feature light computation and on-line processing to obtain real-time filtering. For convenience, we call this algorithm ALRE, for Adaptive Lattice-type Respiratory rate Estimator. In the following subsection, we explain the general transference of approaches from an AR model to an adaptive notch filter, which is the core concept in our method, as well as the relationship between Burg’s algorithm for the AR model and our ALNF structure about their lattice forms.

### Autoregressive modeling method and our proposed method

The AR model is one of many signal modeling techniques, and is composed only of autoregressive and error terms, as follows:
2

where *x*(*n*) is the target signal and *e*(*n*) represents the residual error. *M* and *k*_*j*_ denote the AR model order and coefficients, respectively. The AR modeling procedure means to find the optimal model coefficients *k*_*j*_, which minimizes the energy of the residual error *e*(*n*).

The AR model can be used as a parametric method for spectrum estimation. Spectrum estimators can be classified into two categories, the parametric method and the non-parametric method; their characteristics are explained in [20]. When we have *a priori* knowledge about the signal, we can apply parametric methods and find more accurate estimates by using the known information about the signal. Therefore, it is important to find an optimal model order.

Application of the modeling method can also be extended from spectrum estimation to frequency estimation because the spectrum contains frequency information [20]. In biomedical signal processing fields, the AR modeling method has been frequently used for RR estimation from a PPG [9–11, 14]. The AR modeling method has three steps in total: downsampling, AR modeling, and RR estimation from the AR model coefficients. The AR modeling method requires two parameters, such as a down-sampling frequency and an AR model order, and it is necessary to find optimal parameters. For example, if the down-sampling frequency is 1 Hz, the AR modeling method cannot detect a RR higher than 30 bpm (0.5 Hz). This means that the AR modeling method imposes a constrained range of RR estimates when the down-sampling frequency is already determined. The AR modeling procedures are explained in detail in [10].

The AR method is an attractive technique for RR estimation from a PPG, because (1) its estimate is considerably accurate, (2) it has a simple algorithm structure, and (3) a short data set is sufficient for RR estimation. Furthermore, it can be applied in a real-time implementation by using the moving-window method. However, a sufficient window length is required for stable estimation; therefore, for real-time applications, each window has to be overlapped with the one next to it. As a result, the AR modeling method has an inefficient computational structure in real-time applications, and the estimate of the AR method lags behind the true RR varying with time. These problems are caused by the batch processing and windowing approach. Thus, we suggest an adaptive and recursive method using an adaptive notch filter and compare it with Burg’s method, which is one of the most common AR modeling techniques [20, 21].

**Figure 3:**
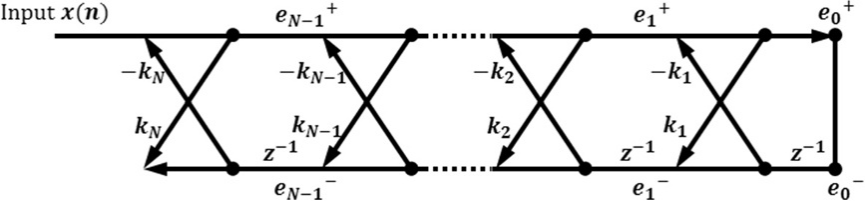
**All-pole lattice filter for Burg’s method.** *e*_*j*_^+^ means j-th forward prediction error and *e*_*j*_^−^ is j-th backward prediction error.

Burg’s AR modeling method is derived from an all-pole lattice filter which has only feedback procedures, as shown in Figure 3. In Figure 3, *k*_*j*_ represents a filter coefficient and, equivalently, an AR model coefficient in Burg’s method. When the model order is *M*, the coefficient *k*_*j* + 1_ is determined by a combination of the *j* th order forward prediction error *e*_*j*_^+^ and the backward prediction error *e*_*j*_^−^, as follows:

3

In this paper, we call Burg’s AR modeling-based RR estimation method as “Burg’s method” for the sake of simplicity.

In this study, we suggest a novel way to estimate RR from PPG using the frequency estimator (ALNF) based on an adaptive notch filter. The adaptive notch filter can estimate the dominant frequency of a given signal. We can intuitively understand the relationship between the AR modeling method and the adaptive notch filter through a simple example. Let us consider a second-order AR modeling problem. A pair of poles of this model is directly linked to a pair of peaks of spectrum, which represents one dominant frequency. Thus, we can estimate the dominant frequency from the model coefficients (a pair of poles). If we invert the transfer function of this AR model, then we can obtain a notch filter and estimate the frequency from a pair of zeroes of the notch filter. Eventually, finding AR model coefficients and designing a notch filter are similar techniques, and we can adaptively trace RR (or HR) by using the adaptive notch filter instead of the AR method. In this study, we chose an ALNF, which is an adaptive IIR notch filter combined with a lattice form and which serves as a frequency estimator in the ALRE. Thus, the ALRE is composed of two ALNFs and a harmonic IIR notch filter. The ALNF and Burg’s method have theoretically similar backgrounds because they are both based on a lattice filter structure.

As we mentioned earlier, the optimal selection of the AR model order is quite important to estimate a spectrum and frequencies. However, unlike the AR modeling method, the adaptive notch filer’s order is essentially fixed at 2, and it estimates only a single dominant frequency. To compensate for this crucial problem, we designed a sequential IIR notch filter, which utilizes the estimated HR as its adaptation parameter to obtain the respiratory component from the PPG.

### The structure of the proposed algorithm: ALRE

The key idea of the ALRE is the sequential isolation of cardiac and respiratory signals; a PPG can be decomposed into its cardiac and respiratory components. The sequential isolation approach is important for the RR estimation because the respiratory and cardiac components are mixed in a PPG at the same time. Considering this concept, we designed the ALRE that includes three processes to estimate HR and RR. The overall structure of the ALRE is shown in Figure 2.

The first step is the estimation of HR using a frequency estimator (ALNF) which traces the fundamental frequency of a harmonic signal. To extract the pure HR component, we employ an IIR band pass filter which has a reasonable pass band (0.5 ~ 5 Hz) based on a feasible HR range prior to HR estimation.

In the second step, the cardiac component (HR and its harmonics components) in the PPG are removed through a harmonic IIR notch filter with an estimated HR. The harmonic IIR notch filter structure is very well-known and commonly used in the signal processing field [22]. Although a previous study [8] showed that it is possible to reduce the cardiac component and enhance the respiratory component by using an IIR low-pass filter, some portion of the cardiac component remain in the respiratory component because the IIR low-pass filter is limited to eliminating the cardiac component whose frequencies are above the cut-off frequency. Thus, we used a harmonic IIR notch filter next to the frequency estimator. The harmonic IIR notch filter is composed of a serial connection of second-order tunable IIR notch filters as follows:
4

**Figure 4:**
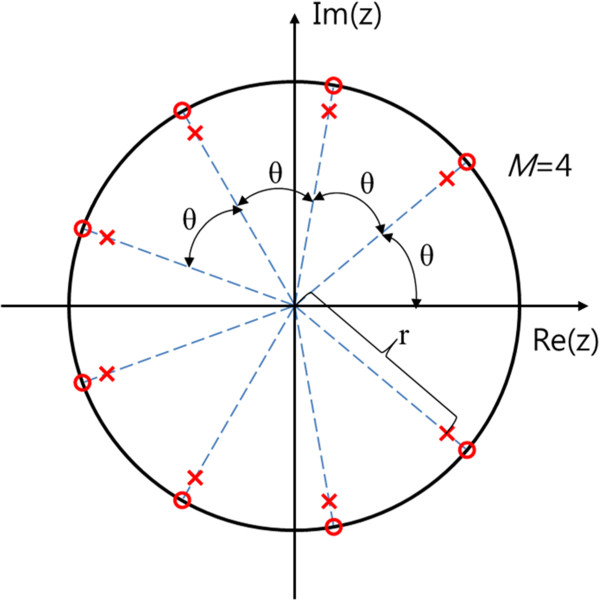
**An example of the pole-zero map of harmonic IIR notch filter with 4 harmonic components.**

Figure 4 shows an example of the pole-zero map of a harmonic IIR notch filter for the removal of four harmonic components (*M* =4). *r* represents the distance between the origin and the pole, and it can control the bandwidth of the notch, which becomes narrower as *r* approaches 1. We chose 0.95 as the value of *r* in the ALRE. In the ALRE, *θ* is particularly assigned to HR, which is estimated by the frequency estimator (ALNF) in the first step. *M* is the number of serial connections, that is, of harmonic components. The frequency response of this filter seems a comb with several notches at the fundamental frequency and its harmonics (not shown here, refer to [22]).

The final step is RR estimation. From the second step, we obtain a residual signal after the cardiac components were eliminated from the PPG through the harmonic IIR notch filter (see Figure 2). Given the residual signal, we performed a band pass filter (0.1 ~ 2 Hz) to refine the respiratory component, by which we can stably estimate RR using another frequency estimator. In this sense, the process of RR estimation is similar to the HR estimation step.

Altogether, the ALRE adaptively eliminates the cardiac signal and its harmonic components from a PPG through HR estimation and sequential harmonic IIR notch filtering, and then it estimates RR from the residual signal (the respiratory component). Thus, unlike the AR method, it does not restrict the estimated HR or RR ranges but uses only the feasible ranges (HR: 0.5 ~ 5 Hz, RR: 0.1 ~ 2 Hz). Within the feasible frequency ranges, the frequency estimators are designed to find the target frequencies (HR and RR). In this study, we adopt the ALNF as a frequency estimator and briefly review the ALNF in the next subsection.

### Adaptive lattice notch filter - review

**Figure 5:**
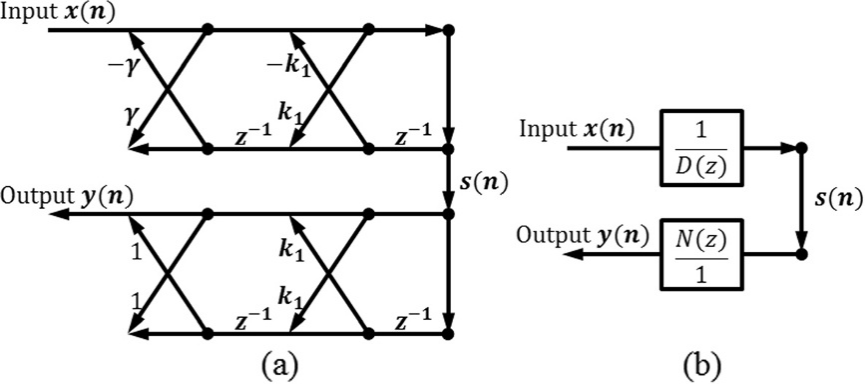
**IIR lattice notch filter structure for ALNF.** Upper part means all-pole filter and lower part is all-zero filter. **(a)**: signal flow graph representation, **(b)**: block diagram representation. *D*(z) and *N*(z) represent denominator and numerator of transfer function of IIR lattice notch filter, respectively.

The ALNF method was initially proposed in [18, 19]. It is an adaptive notch filer combined with a lattice form for an adaptation algorithm. Initially, its IIR filter structure is separated into all-pole and all-zero filters (see Figure 5):

56

where *x*(*n*) and *y*(*n*) represent input and output signals, respectively. *s*(*n*) represents the output of the initial all-pole filter part, and *k*_1_ is the filter’s adaptation parameter. *γ* corresponds to the pole and zero contraction factor. Eqs. (5) and (6) represent all-pole and all-zero filtering processes, respectively. By minimizing E[*y*^2^(*n*)], we can get the value of the adaptation parameter *k*_1_:
7

where *R*_*s*_(0) and *R*_*s*_(1) are the autocorrelations of *s* (*n*), which should be calculated by a statistical process. To alternatively realize the statistical process in real-time, the weighted least-square algorithm can be used: , where *y*^2^(*j*) and *w*_*n* − *j*_ represent an output power of the ALNF and a weight parameter, respectively. The weighted least-square algorithm can be arranged by recursive processes using the forgetting factor *η* [23]:
8910

Furthermore,  is clipped to prevent divergence:
11

In addition, in order to sustain the stable state, a smoothing process is conducted using the smoothing factor *μ*.
12

where  is the estimate of *k*_1_. Given , we can estimate the frequency 13

Thus, ALNF has three parameters:  (pole and zero contraction factor), *μ* (smoothing factor), and *η* (forgetting factor).  in Eq. (6) represents the contraction between pole and zero (0 <  <1), so that it is matched to the sharpness of the frequency response of the filter. When  is closer to 1, the notch will become narrower. The smoothing factor *μ* is designed to enhance the stability, and the forgetting factor *η* represents the update parameter for the recursive form of *R*_*s*_ (autocorrelation of *s* (*n*)). Each parameter’s characteristics and the algorithm’s configuration are explained in detail in [18].

The ALNF, which was adopted as frequency estimators in ALRE is theoretically a single-tone frequency estimator [18, 19], but the adaptive IIR notch filer is generally robust to sinusoidal noise (colored noise) [24, 25]. Further, the ALNF is less computationally demanding [26, 27]. Despite these advantages, its estimate can be biased when the colored noise is added to the input signals. Because a PPG has several frequency components, the colored noise interference commonly occurs and biased estimation is inevitable. However, the amount of bias of the ALNF is considerably small. The characteristics of the ALNF are discussed below.

### Analysis of the ALNF

Before using the ALNF, it is necessary to analyze the ALNF regarding its bias characteristic. Originally, the ALNF was designed to estimate the frequency of a single tone, and it is theoretically unbiased when the input is a single sinusoidal with additive white Gaussian noise [18].

To analyze the baseline modulation, we consider a two-tone signal *x* (*n*) with additive white Gaussian noise *v* (*n*):
14

where *ω*_0_ and *ω*_1_ are constant frequencies and *ω*_0_ ≠ *ω*_1_, the phases (*ϕ*_0_ and *ϕ*_1_) are mutually uncorrelated random variables, and *R*_*s*_ (*k*) is calculated as follows:
15

As a result, the ALNF traces the biased frequency
1617

**Figure 6:**
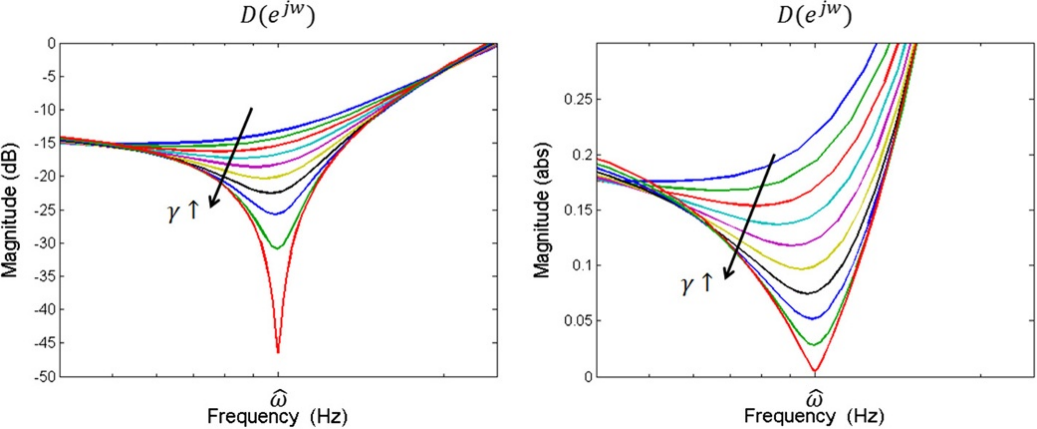
**Frequency response for the inverse of all**-**pole filter part of the IIR lattice notch filter.** Vertical axis unit of left-side is dB and that of right-side is absolute value.

As in Eq. (16) and Eq. (17), the bias is theoretically generated by the addition of a sinusoidal signal and can be determined by the frequency response of the all-pole filter part . As can be seen in Figure 6, *D*(*e*^*jω*^) is similar to the frequency response of the notch filter; its shape is controlled by *γ* in Eq. (5) and its center frequency is  in Eq. (17). When  asymptotically reaches 1, the frequency response goes to the notch, and the gain of the center frequency approximately becomes 0. For example, if *ω*_0_ is dominant frequency component with large *A*_0_, then  (center frequency of *D*(*e*^*jω*^)) approaches nearby *ω*_0_ and *k*_1_ ≈ − *cos*(*ω*_0_) because  will be approximately 0 but not equal to 0. Identically, if *ω*_1_ is dominant frequency component, then  reaches some frequency close to *ω*_1_ and *k*_1_ approximately becomes − *cos*(*ω*_1_). In this case,  can approximately converge into *ω*_1_, and its accuracy depends on *D*(*e*^*jω*^) on *ω*_0_ or *ω*_1_. As a result, we can ensure that large values of *γ* decrease the bias of the ALNF to the sinusoidal interference or colored noise.

### Comparison of performance using simulated signals

**Figure 7:**
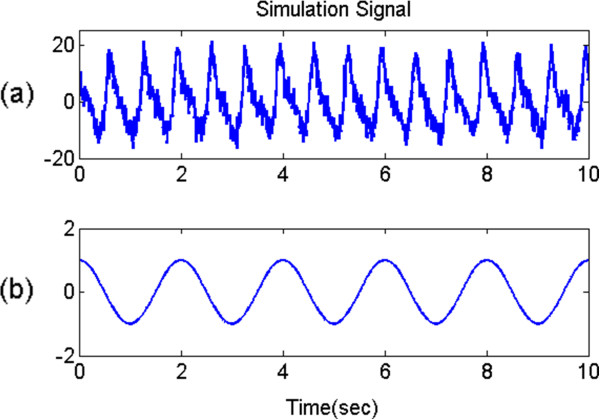
**Simulation signal. (a)**: PPG with white Gaussian noise (20 dB), **(b)**: reference respiratory signal (constant frequency).

To compare the algorithms’ performance, we executed a rigorous simulation. All the procedures were implemented in Matlab®. First, we constituted an input signal *x* (*n*) as follows:
18

where *x*_*cardiac*_(*n*), *x*_*resp*_(*n*), and *ν*(*n*) represent the cardiac signal, respiratory signal, and white Gaussian noise, respectively. *f*_*HR*_ and *f*_*RR*_ represent the HR and RR, and particularly *f*_*RR*_ corresponds to the first derivative of *ψ*(*n*) which is an operand of the cosine function of *x*_*resp*_(*n*). *f*_*s*_ is the sampling frequency. We set *x*_*cardiac*_(*n*), which was modeled based on the cardiac components of the PPG, with HR (*f*_*HR*_). Furthermore, to cover various situations, three types of RR (*f*_*RR*_), such as a single sinusoidal with constant frequency (for normal situations), a linear chirp (for urgent situations) and a sinusoidal frequency modulation (FM) signal (for exercise situations), were used for respiratory component modeling in the PPG. By adding *x*_*cardiac*_(*n*) and *x*_*resp*_(*n*), we constructed the simulated PPG signal *x*(*n*), and its shape was similar to the real PPG (see Figure 7). From the simulation signals, RR estimation was performed by the ALRE and Burg’s method. For the comparison between these two algorithms, we found optimal parameters for each algorithm, which minimize root mean square error (RMSE). We adopted a 30-s moving window for Burg’s method, and for the real-time estimation, each window was overlapped by 25-s duration with the neighboring windows. By the moving window method, the RR estimated by Burg’s method was updated at a 5-s interval.

**Figure 8:**
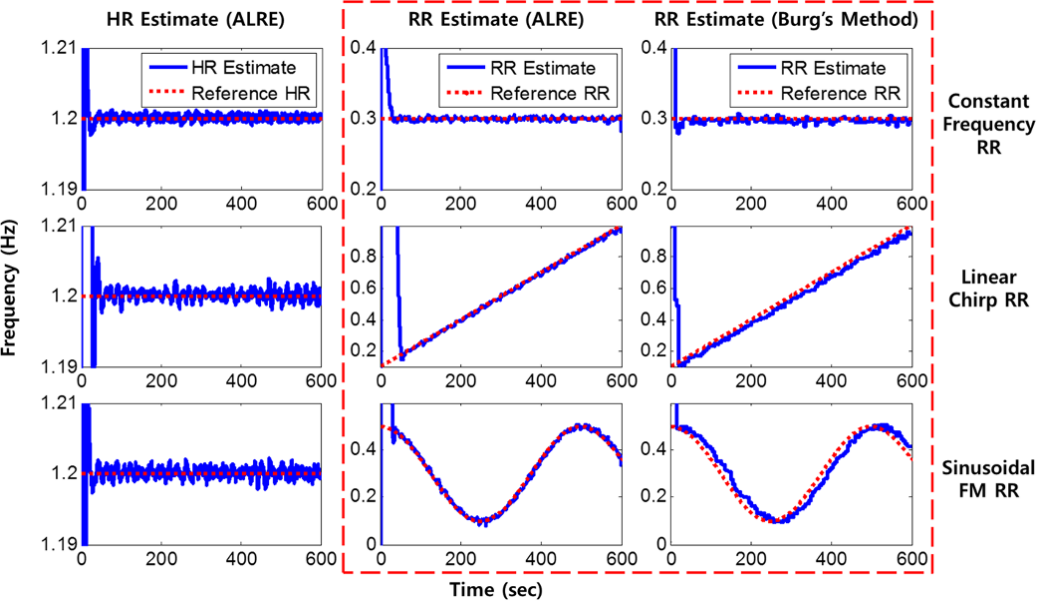
**RR tracking from simulated PPG.** Solid lines represent estimates and dotted lines are reference values when all input signal’s SNRs are 10 dB. Dashed red box includes only RR estimation results for the ALRE and Burg’s method.

The estimation of RR by the ALRE method showed better performance than Burg’s method and the result is compared in the dashed box of Figure 8. The HR and RR estimated by the ALRE are depicted in the first and second columns, respectively. The third column demonstrates the result of Burg’s method for RR estimation. The first row of Figure 8 illustrates the estimation result when the respiratory signal is modeled by the constant frequency single sinusoidal signal. The second and last rows depict the results when the respiratory signals are simulated by the linear chirp signal and the sinusoidal FM signal, respectively. The dotted lines in Figure 8 represent true reference frequencies and the solid lines indicate values estimated from PPG. From the estimated frequencies, we calculated the estimation error between the true and estimated values when each method has an optimal parameter, and the results are summarized in Table 1. Because of the initial convergence time, all RMSEs were calculated from 80 s to the end. With the simulated signals, the ALRE generally shows better performance than Burg’s method as shown in Table 1 and Figure 8.

**Table 1:** **RMSEs of RR estimation under simulated signal**

	Constant frequency		Linear chirp		Sinusoidal FM	
	Burg’s method	ALRE	Burg’s method	ALRE	Burg’s method	ALRE
	(***df***_***s***_, order)	( ***η***, ***μ***)	(***df***_***s***_, order)	( ***η***, ***μ***)	(***df***_***s***_, order)	( ***η***, ***μ***)
**0dB**	0.005350	0.002204	0.148526	0.023145	0.093723	0.013340
	(1 Hz, 10)	(0.999, 0.989)	(2 Hz, 16)	(0.998, 0.983)	(1 Hz, 7)	(0.996, 0.996)
**5dB**	0.003908	0.001567	0.149505	0.008220	0.046544	0.008827
	(1 Hz, 8)	(0.999, 0.987)	(2 Hz, 15)	(0.996, 0.983)	(1 Hz, 3)	(0.997, 0.981)
**10dB**	0.002030	0.001106	0.088621	0.006885	0.031328	0.006998
	(1 Hz, 9)	(0.999, 0.983)	(3 Hz, 4)	(0.980, 0.996)	(2 Hz, 12)	(0.996, 0.98)

According to simulation signal types and SNRs, RMSEs of each method are listed and the optimal parameter values are placed in each round bracket. (*df*_*s*_: down sampling frequency, order: AR model order, *η*: forgetting factor, *μ*: smoothing factor).

The ALNF method has three kinds of parameters (*γ*: pole and zero contraction factor, *μ*: smoothing factor, and *η*: forgetting factor) that determine the performance of the ALRE method when the signal is given. Therefore, it is necessary to search for optimal parameter values to minimize the estimation error. As mentioned before, the robustness to colored noise was enhanced as γ increased. Therefore, we set *γ* sufficiently close to 1. The other parameters were also assigned to large values because the error generally decreased as *μ* (smoothing factor) and *η* (forgetting factor) were incremented. We confirmed the better results of RR estimation with higher values of varying *μ* and *η* as well as γ from the three simulation datasets.

### Data collection

**Figure 9:**
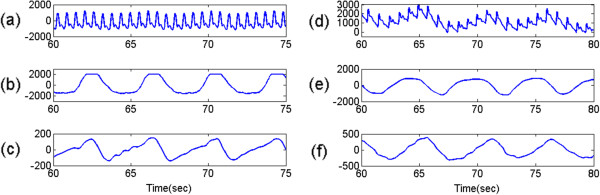
**Experiment data. (a)** and **(b)** represent PPG and the reference respiratory signal from MIT MIMIC data, respectively. **(d)** and **(e)** are PPG and the reference respiratory signal measured by BIOPAC® device. **(c)** and **(f)** depict residual signals obtained by the ALRE approach from MIT MIMIC data and measured signal, respectively. The unit of vertical axis is mV.

Initially, we used an open-source data set, and additionally, our own PPG and respiration signals measured by a BIOPAC® device. The open-source data was adopted from the MIT MIMIC Database, which has been used in previous studies [28]. We particularly isolated 50 data sets that were less contaminated by artifacts, and these were used for assessing the performance of the RR estimation. Each data set was recorded at a sampling rate of 125 Hz for 9 min 40 s, and it included the PPG and (reference) respiratory signal (see Figure 9). In addition, we collected PPG and respiratory signals from five male and one female subjects (Age = 28.7 ± 1.9 years). Each subject was instructed to take a breath randomly but without limiting RR during four trials of data acquisition (Figure 9). In total, 24 trials were collected, and in each trial, data was recorded with a 125-Hz sampling rate for 5 min using a BIOPAC® PPG100c and RESP100c. In order to calculate the estimation error, the reference RR was evaluated by the zero crossing method from the given respiratory signal.

## Results

**Figure 10:**
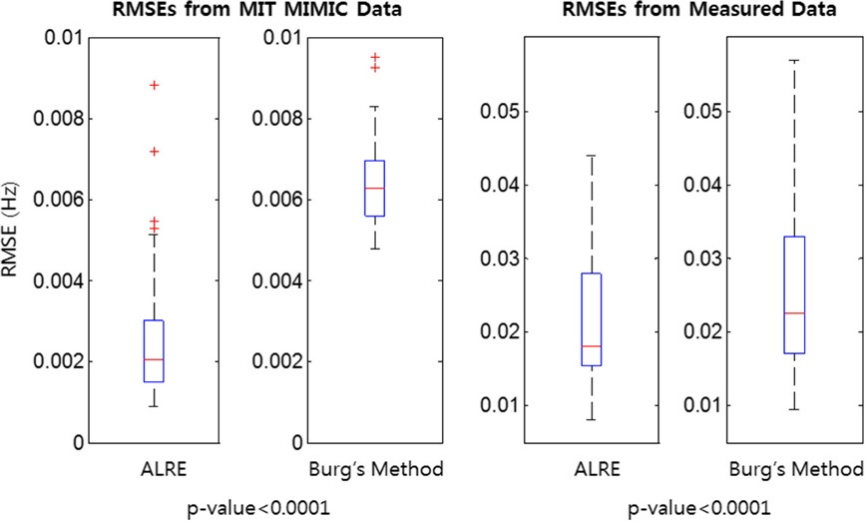
**Distribution of RMSEs of RR estimation.** Upper and lower boxes represent the distribution of RMSE from 25th to 75th percentiles. Center, top, and bottom line indicate 50th, 90th, and 10th percentiles. Left two columns means RMSEs (the ALRE’s and Burg’s method’s) from MIT MIMIC data, and right two columns represent RMSEs (the ALRE’s and Burg’s method’s) from experiment data measured by BIOPAC® device. P-values of paired t-test for MIT open source data and Wilcoxon signed rank test for measured data are less than 0.0001.

For the verification of the ALRE’s performance, the RMSE was calculated by the difference between the reference RR and the RR estimated from the PPG when each method’s parameters had optimal values to minimize error. All RMSEs were calculated from 80 s to the end. To compare the two methods, we performed statistical tests on the MIT open-source data by using a paired t-test and on our measured data with non-parametric Wilcoxon’s two-sampled signed rank test under calculated RMSEs; the p-values of both tests were less than 0.0001. As shown by the two statistical tests, the ALRE’s RMSEs were significantly smaller than those of Burg’s method, which means the ALRE was superior to the conventional Burg’s method for RR estimation from a PPG. Figure 10 represents box plots of RMSEs for the ALRE and Burg’s method applied to the MIT open-source data and our experimental data measured by the BIOPAC®. In Figure 10, the upper and lower boxes represent the 75th and 25th percentiles, respectively, and the center, top, and bottom lines indicate the 50th, 90th, and 10th percentiles, respectively. Asterisks represent outliers in the RMSE distribution. Although we can get an additional HR estimate during the RR estimation process of the ALRE procedure, we did not directly assess HR estimation. However, it could be indirectly evaluated by the result of the RR estimation because the RR estimation process of the ALRE contains the HR estimation. Thus, we calculated the estimation error of RR with the actual data only.

**Figure 11:**
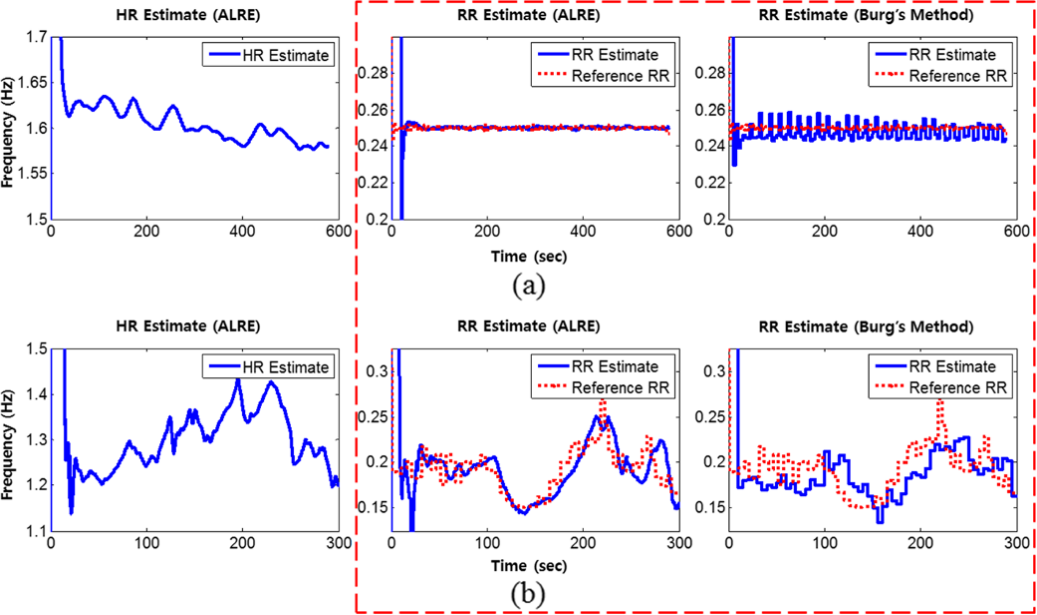
**RR tracking from real data. (a)**: HR and RR tracking from MIT MIMIC data, **(b)**: HR and RR tracking from measured data. Solid lines represent HR and RR estimates and dotted lines are reference RR values. Dashed red box includes only RR estimation results for the ALRE and Burg’s method.

As a result, the ALRE showed more accurate estimation results than Burg’s method did. In Figure 11, the dotted line represents the reference RR, which was acquired from the respiratory signal, and the solid lines correspond to the RR and HR estimates. Figure 11 shows that the ALRE’s tracking performance was superior to Burg’s method, and the variances of estimates were also smaller than those for Burg’s method.

## Discussion

Because a PPG contains both cardiac and respiratory components, many methods have been proposed to obtain a RR estimate from a PPG [5]. Although it is possible to estimate RR from PPG, the process is difficult because the respiratory component in a PPG is not as easily observed as the cardiac component. Therefore, previous methods, such as VFCDM and the AR modeling method, required complicated batch processes, which imply an inefficient computation structure for real-time applications [10, 12–14].

Because of the development of sensing and communication techniques, the healthcare industry has made much progress in recent years; to further enhance this progress, mobility and fast processing are increasingly in demand [15]. To meet those requirements, we set design conditions, light computation and on-line processing. Considering these conditions, we propose the ALRE algorithm. The ALRE is an on-line processor that is constructed by an adaptive and recursive algorithm. Through a sequential process, a PPG can be decomposed into its cardiac and respiratory components. With the decomposed signals, the ALRE can estimate HR as well as RR using the ALNFs, which feature light computation and robustness to interfering input sinusoids. The ALNF has three parameters: a pole-zero contraction factor, a forgetting factor, and a smoothing factor, which characterize the ALNF [18, 19]. Before applying the ALNF to the proposed ALRE, we searched for appropriate values of the ALNF’s parameters through mathematical analysis and simulation; furthermore, we assessed the ALRE algorithm with real data. Our approach showed not only simple on-line processing (light computational burden) but also high estimation accuracy compared to Burg’s method.

Although HR has its own variation, our algorithm accurately traced the cardiac component and removed it by harmonic IIR notch filter. ALRE estimated RR accurately and it was very close to the reference RR as in the middle column of Figure 11 in contrast to Burg’s method (right column of Figure 11). The respiratory components acquired after harmonic IIR notch filtering are presented in Figure 9 (c) and (f).

Burg’s method with model order *M* generally has an computational cost per iteration as *O*(*M*^*2*^) [29] and our ALRE with harmonic IIR notch filter order *M* has *O*(*M*) [30]. In terms of analysis, to compare between them meticulously is a little difficult so that we measure the computation time using real data with 9 min 40 s duration. That is, the computation time of ALRE was 0.0548 s much shorter than those of Burg’s method which had different overlapping durations with neighboring windows. As the overlapping increased, the Burg’s method with *M* = 6 showed increasing computation times with 0.1810 s (overlapping: 0 s), 0.2205 s (5 s), 0.2739 s (10 s), 0.3808 s (15 s), 0.5883 s (20 s), and 1.2076 s (25 s). Consequently, the computational load of Burg’s method depends on the overlapping duration and ALRE takes less computational burden than Burg’s method.

ALRE does not restrict the ranges of the HR or RR estimates, whereas the AR modeling method has a restriction on its frequency estimation range caused by the down-sampling frequency [10, 11]. For example, the ALRE can cover the physiologically feasible ranges of HR from 0.5 to 5 Hz and RR from 0.1 to 2 Hz, which cannot be implemented by the AR method with a down-sampling frequency under 4 Hz [10, 11]. Taken together, the ALRE might be considerably better than Burg’s method in real-life and real-time applications.

Although the ALRE has several advantages, it still should be improved to be embedded in mobile devices because of the problem of convergence time. The initial convergence time of the ALRE is not a critical problem, but a short transient time can enhance the tracing performance for varying HR and RR situations. In fact, convergence time can be controlled by the forgetting factor and smoothing factor. If these two parameters have smaller values, then convergence time will possibly be shorter. However, smaller parameter values result in worse performance. Therefore, we should consider a trade-off between convergence time and the fidelity of estimation. In order to break through this limitation, we are planning to investigate a faster and more precise frequency estimator or an adaptive parameter updating strategy for future work.

## Conclusions

In conclusion, the novelty of the ALRE stands out because of its simple structure and fast processing without constrained ranges of HR or RR estimate. Therefore, it can contribute to daily cardiopulmonary system monitoring. Even though fast HR and RR tracking remain to be improved, the proposed ALRE approach can substitute for the AR modeling method for RR estimation. In addition, we expect this algorithm to be applied to other physiological signals that contain several health conditions at the same time, such as a mixed signal composed of a fetal heartbeat and respiration.

## Abbreviations

PPG: Photoplethysmogram
HR: Heart rate
RR: Respiratory rate
AR: Autoregressive
IIR: Infinite impulse response
ALNF: Adaptive lattice notch filter
ALRE: Adaptive lattice-type RR estimator
RMSE: Root mean square error.
